# Bioinformatic Detection of Positive Selection Pressure in Plant Pathogens: The Neutral Theory of Molecular Sequence Evolution in Action

**DOI:** 10.3389/fmicb.2020.00644

**Published:** 2020-04-09

**Authors:** Mark C. Derbyshire

**Affiliations:** Centre for Crop and Disease Management, School of Molecular and Life Sciences, Curtin University, Perth, WA, Australia

**Keywords:** dN/dS, selective sweep, plant pathogen, effector, positive selection

## Abstract

The genomes of plant pathogenic fungi and oomycetes are often exposed to strong positive selection pressure. During speciation, shifts in host range and preference can lead to major adaptive changes. Furthermore, evolution of total host resistance to most isolates can force rapid evolutionary changes in host-specific pathogens. Crop pathogens are subjected to particularly intense selective pressures from monocultures and fungicides. Detection of the footprints of positive selection in plant pathogen genomes is a worthwhile endeavor as it aids understanding of the fundamental biology of these important organisms. There are two main classes of test for detection of positively selected alleles. Tests based on the ratio of non-synonymous to synonymous substitutions per site detect the footprints of multiple fixation events between divergent lineages. Thus, they are well-suited to the study of ancient adaptation events spanning speciations. On the other hand, tests that scan genomes for local fluctuations in allelic diversity within populations are suitable for detection of recent positive selection in populations. In this review, I briefly describe some of the more widely used tests of positive selection and the theory underlying them. I then discuss various examples of their application to plant pathogen genomes, emphasizing the types of genes that are associated with signatures of positive selection. I conclude with a discussion of the practicality of such tests for identification of pathogen genes of interest and the important features of pathogen ecology that must be taken into account for accurate interpretation.

## Introduction

The theory underpinning the evolution of nucleic acid sequences can be traced all the way back to *On the Origin of Species by Means of Natural Selection* (Darwin, 1859), which was published in 1859. In this seminal work, Charles Darwin lays down the fundamental tenets of his theory of natural selection. There are three basic principles that form the core of this theory: (1) each generation produces random deviations from the parental form; (2) random deviations can be either advantageous, disadvantageous or neutral; and (3) individuals with advantageous differences to their parents are more likely to reproduce, those with disadvantageous differences less so, and those with neutral or no differences equally as likely.

The phenotype of an individual is controlled by its DNA sequence. Differences between phenotypes are caused by differences in the DNA sequence that affect some aspect of gene expression. The term for these differences is ‘alleles,’ with the possibility of each of the four nucleotides and deletions, insertions, or rearrangements occurring at each of the sites in the genomes of different individuals in a population. Differences in phenotype can also be caused by chemical modifications of the DNA sequence and its related regulatory proteins, so called ‘epigenetic’ changes (Cavalli and Heard, 2019). The deviations in phenotype between individuals in each generation are caused by inheritance of different combinations of alleles from parental genotypes and spontaneous mutations in germ-line cells. Alleles that cause advantageous phenotypes with a greater reproductive rate are said to be under positive selection. Those that cause disadvantageous phenotypes with poorer reproductive rates are said to be under negative selection. Other alleles that have no impact on phenotype (or impacts that do not affect reproductive rate) are said to be neutral (Molles and Sher, 2018).

In the last half a century or so, many studies have explored the effects of natural selection on appearance and spread of new alleles in populations. It was in this time that one of the founding theories of modern genetics was developed by Motoo Kimura in 1968: the neutral theory of molecular evolution (Kimura, 1968). This paper develops the hypothesis that most mutations are selectively neutral. Therefore, the majority of variation between individuals in populations consists of alleles that are not undergoing positive or negative selection. It follows that most fixed differences between species or divergent populations are caused by a phenomenon called ‘genetic drift.’ This is the random spread of neutrally selective alleles through a population to reach fixation without influence from selective pressure (Masel, 2011). Many of the major statistical tests for positive selection in DNA sequences use the neutral model as a null hypothesis. Although not without criticism, correct application of tests such as these has provided profound insights into the evolution of many different species.

Some species naturally attract the attention of biologists, due to their interesting evolutionary dynamics, economic or societal importance, or ease of genetic manipulation. Two widely researched groups of organisms that fit these criteria are the plant pathogenic fungi and oomycetes. These organisms have been co-domesticated and spread with many of the world’s most valuable crops, and they continue to cause persistent economic losses worldwide (Savary et al., 2019). For instance, the fungus *Fusariam oxysporum* f. sp. c*ubense* causes the infamous ‘Panama disease’ of banana. This disease threatens a large part of the world’s banana crop due to a single virulence gene that facilitates infection of the predominantly grown banana clone Cavendish (Ordonez et al., 2015).

Plant pathogens often undergo strong selective pressures that rapidly change depending not only on the vagaries of the ecosystems they inhabit but also direct inputs from humans. For example, consistent application of fungicides selects for fungicide resistance. Many novel fungicides that inhibit one or a few enzymes found only in fungi have been introduced since the 1970s. At first, these fungicides offered a major boost to agricultural production, as they are highly effective at low doses and minimally toxic to plants and consumers. However, since these initial successes, a diverse array of plant pathogens affecting agricultural production have become resistant to them (Lucas et al., 2015). Due to their enzyme specificity, a single target site mutation that modifies protein structure to mitigate fungicide binding can undergo strong positive selection to become fixed in a pathogen population (Hahn, 2014). This issue is exacerbated by a hostile regulatory environment that diminishes the availability of different modes of action (Lucas et al., 2015).

Another means of combatting plant pathogens is to use crop cultivars with resistance to disease. Some diseases only affect a single plant species, which leads to pronounced antagonistic co-evolution between host and pathogen. This co-evolutionary dynamic is classically described as an interplay between genes in host plants known as ‘R’ (for Resistance) genes and ‘Avr’ (for Avirulence) genes in pathogens (Jones and Dangl, 2006). Pathogen Avr genes initially arise in populations as genes encoding ‘effectors,’ which are proteins with the ability to manipulate the host immune system. This property of effectors allows the pathogen to proliferate in plant tissues undetected. R genes in plants evolve as a recognition system for specific pathogen effectors. In agricultural plant populations, which are typically dominated by a few cultivars, the presence of one or a few specific R genes exerts strong selective pressure on pathogens to lose their corresponding Avr genes and regain pathogenicity via the expression of other effectors. There are several instances of different pathogens overcoming crop cultivar resistance based on R gene guided breeding (Kiyosawa, 1982; Leach et al., 2001). This poses an acute problem for plant breeders as introgression of specific genetic loci typically takes several years (Poland and Rutkoski, 2016).

With development of new sequencing technologies, there has been a large increase in the amount of plant pathogen genomes available for evolutionary studies. Statistical procedures for the detection of selective pressure are now applicable to whole genome data, which aids identification of alleles that may be of major significance to our understanding of plant pathogen biology. The aim of this paper is to discuss the basic principles of two commonly used tests for directional selection that use neutral sequence evolution as the null hypothesis – the *d*_*N*_/*d*_*S*_ ratio test and the selective sweep scan. The former is applicable when considering extended evolutionary trajectories spanning speciation events whereas the latter is appropriate when considering population samples within species. The application of these two types of tests to plant pathogens in the post-genome sequencing era will be discussed with an emphasis on the kinds of genes identified and the incompatibility of some tests with reproductive lifestyles of some pathogen species. I limit this article to positive selection, excluding balancing and negative selection, as most plant pathogen studies aim at identifying rapidly evolving positively selected genes. Indeed, the selective sweep scan is generally limited to detection of alleles under recent strong positive selection.

## A Brief Explication of the *d*_*N*_/*d*_*S*_ Ratio Test and Its Main Assumptions

The *d*_*N*_/*d*_*S*_ ratio, usually expressed as the Greek symbol ‘ω,’ is a metric designed to assess the rate of non-synonymous nucleic acid changes to the rate of synonymous nucleic acid changes in coding DNA sequences. This metric arises from the neutral theory of sequence evolution described by Kimura (1968, 1977). The main assertion of this theory is that random fixation of alleles causes most observed interspecific genetic diversity. For simplicity, the theory considers a total mutation rate of *V*_*T*_, which includes neutral, nearly neutral and deleterious mutations. It does not include advantageous alleles, which are assumed to be rare. To estimate the rate of fixation of mutant alleles in the population, *K*, the following formula is derived:

$$K=V_{T}f_{0}$$

Where *f*_0_ is the fraction of mutations that are selectively neutral or nearly neutral. Thus, the fraction of total mutations that are not deleterious is what generally determines the rate of nucleotide fixations between divergent lineages.

The foundation of the *d*_*N*_/*d*_*S*_ ratio test for selective pressure was presented not long after Kimura’s explication of the neutral theory. Miyata and Yasunaga (1980) developed a formula to estimate rates of non-synonymous and synonymous changes over time. This estimation is based on analysis of the potential changes that have occurred between two homologous codons from different species.

A codon, denoted α, that undergoes a nucleotide substitution becomes a different codon, α′. If the different codon encodes the same amino acid, it is defined as a ‘synonymous change’ and if it encodes a different amino acid, it is defined as a ‘non-synonymous change.’ For each codon, there are three possible nucleotide changes. In most cases, changes in the first and second positions always cause amino acid changes. However, due to the level of degeneracy in the genetic code, third positions may have different numbers of substitutions that would lead to a synonymous change in the codon. For example, in a fourfold degenerate codon, the three substitutions that are possible in the third position all lead to synonymous changes in the codon. However, in twofold degenerate codons, there are two potential substitutions that cause a non-synonymous change in the codon and one that causes a synonymous change. The fraction of possible substitutions that cause synonymous changes for the *i*^*th*^ position in the codon is defined as *f*_α*i*_. Thus, the fraction of possible substitutions that cause a non-synonymous change is 1 − *f*_α*i*_. These fractions are then applied per codon to estimate the number of synonymous sites, *V*_*S*_, and the number of non-synonymous sites, *V*_*A*_, as follows:

$$v_{S}=\sum_{i=1}^{3}f_{\alpha i},v_{A}=3-v_{S}$$

This provides a normalization for estimation of the relative rates of synonymous and non-synonymous sites. To illustrate this estimation, the example used in this paper is as follows:

path 1:UUU(phe)↔GUU(val)↔GUA(val)

path 2:UUU(phe)↔UUA(leu)↔GUA(val)

Here, we consider changes from one codon, denoted α, to another denoted β. From the codon *UUU* (α), which encodes phenylalanine, to the homologous codon *GUA* (β)(and vice versa), which encodes valine, there are two possible paths. Path 1 assumes one non-synonymous first position change followed by a synonymous third position change; path 2 assumes a non-synonymous third position change followed by another non-synonymous first position change; a path is denoted as *p*(α,β). The following assumptions are made about the sequence of events leading to evolutionary change from codon *UUU* to codon *GUA*. (1) The differences between the two homologous codons are caused by a total amount of mutations equal to the minimum substitution number (MSN), that is, there have been no back-mutations or double mutations of the same sites; (2) it is unlikely that the two possible paths have equal probability. To account for the second assumption, a weighting factor, ω, for each path is introduced based on whether changes are synonymous, or, if non-synonymous, the degree of similarity between the properties of the amino acids that they encode. Those encoding similar amino acids have a higher weight than those encoding more divergent amino acids.

Extrapolating from the simple estimation of *v*_*S*_ and *v*_*A*_ on a per codon basis, their average values are estimated for a given path as follows:

$${\bar{v}}_{S},p\left(\alpha ,\beta \right)=\frac{\left(v_{S},\alpha +v_{S},\gamma \left(p\right)+v_{S},\delta \left(p\right)+v_{S}\beta \right)}{\left(MSN+1\right)}$$

$${\bar{v}}_{A},p\left(\alpha ,\beta \right)=\frac{\left(v_{A},\alpha +v_{A},\gamma \left(p\right)+v_{A},\delta \left(p\right)+v_{A}\beta \right)}{\left(MSN+1\right)}$$

Where the intermediate codons in the path are γ(*p*) and δ(*p*). For any given path between α and β, there are a total of *MSN* + 1 different codons in the path including α and β. Therefore, to find the average, the total possible number of each type of substitution through the codons in the path is divided by this number.

To then find the expected number of synonymous changes from codon α to codon β, *n*_*S*_(α,β), the weight for each path, ω_*p*_(α,β), is applied to the average number ${\bar{v}}_{S},p\left(\alpha ,\beta \right)$ for the paths and all paths are summed over; likewise for the expected number of non-synonymous changes, *n*_*A*_(α,β).

$$n_{S}\left(\alpha ,\beta \right)=\sum_{p}^{L}\omega_{p}\left(\alpha ,\beta \right){\bar{v}}_{S},p\left(\alpha ,\beta \right)$$

$$n_{A}\left(\alpha ,\beta \right)=\sum_{p}^{L}\omega_{p}\left(\alpha ,\beta \right){\bar{v}}_{A},p\left(\alpha ,\beta \right)$$

For all homologous sites in a sequence, total possible synonymous, *N*_*S*_, and total possible non-synonymous, *N*_*A*_, substitutions can be calculated by summing over all *n*_*S*_ and *n*_*A*_. Then, counting the number of actual synonymous, *M*_*S*_, and non-synonymous, *M*_*A*_, substitutions for each pair of homologous codons (again, weighted and summed across possible paths), the number of each type of mutation can be normalized to the number of possible mutations per site as follows:

$$K_{S}=M_{S}/N_{S}$$

$$K_{A}=M_{A}/N_{A}$$

The ratio of *K*_*A*_ to *K*_*S*_ (which gives rise to the similar *d*_*N*_/*d*_*S*_ statistic in future literature) which is often denoted ω, may then be used to estimate the degree of positive selection acting on a particular gene. Assuming only neutral mutation of the gene, the null hypothesis, one would expect synonymous and non-synonymous fixations to occur at parity. This hypothesis is rejected if there is a high or low ω. If ω is low, there have been fewer non-synonymous fixation events than expected under a neutral regime; if it is high, there have been more non-synonymous fixation events than expected. Since it is the non-synonymous changes that shape protein function, one would infer under the latter scenario that there have been multiple instances of positive selection on changes in protein structure. Under the former scenario, one would infer that the protein structure and function are highly conserved and that any amino acid changes are quickly lost through negative selection.

However, there are major limitations to this test that render it too insensitive for practical application. First, it averages *d*_*N*_ and *d*_*S*_ across all sites in the alignment. Therefore, to reach an ω > 1 a protein would have to undergo a large number of adaptive changes throughout its sequence that reach fixation. In reality, adaptive mutations are often limited to particular sites in the enzyme, which confer the main part of its function. Second, this model also assumes the same ω along each lineage in the alignment. Thus, the average ω throughout lineages under investigation would have to be high enough to detect selective pressure. This may not be the case if, for instance, only a single lineage underwent multiple adaptive fixations.

Thus, several tests have been developed to account for variation in ω between sites and lineages. The main principle of these tests is the use of maximum likelihood models that either only describe neutral and deleterious mutations or allow for positive selection. To consider only site specific adaptive mutations, a standard likelihood ratio test can be performed between a model that includes only a parameter for selectively neutral or deleterious mutations and a model that extends this parameter to include a class of sites that have been positively selected (Nielsen and Yang, 1998). Extending this model, one can consider whether positive selection has been active in a given lineage by treating it separately to the rest of the lineages in a phylogenetic tree. The lineage under investigation is the ‘foreground’ lineage whereas the rest of the lineages in the tree are the ‘background’ lineages. The null model presumes that no sites have undergone positive selection in either background or foreground lineages. The alternate model extends a parameter to include positive selection in the foreground lineage. Again, a standard likelihood ratio test can be performed to assess whether the extended model fits better than the null model (Yang and Nielsen, 2002). These maximum likelihood tests for directional selection are implemented in the phylogenetic package PAML (Yang, 1997). For a detailed description of how to apply these tests to whole genomes, the reader is directed to the book chapter by Jeffares et al. (2015).

A final point about the *d*_*N*_/*d*_*S*_ ratio test is that its implicit assumption is that one is considering alignments of nucleotide sequences of individuals from populations that have undergone multiple independent fixation events. The relative substitution rates are assumed to represent fixation events in the divergent sequences that have occurred either through genetic drift or directional selection. For most organisms, such events are most likely to occur on extended time scales spanning speciation events. Despite this, many studies have applied the ω test to alignments of mRNAs from individuals in a population of the same species.

Simulation studies have shown that if the assumption of sufficient divergence between the sequences is broken, the ω calculated for the two sequences cannot be interpreted in the way originally intended. The main issue arises when selective pressure on the sequence is high. Within a population, sequences of a gene that is undergoing a selective sweep are much less diverse than others. This is because the same favorable allele has been passed through most individuals in the population, bringing with it all alleles either side that are too close to be lost through recombination (Kryazhimskiy and Plotkin, 2008). Also, a high rate of intraspecific non-synonymous mutations that have not been fixed within a population is more suggestive of balancing selection than positive selection. This is a situation where a protein is subjected to heterogenous selection pressures throughout the species’ range. This would lead to favoring of different protein sequences depending on local environment. This is not to say that application of ω based tests within species is incorrect, just that under these circumstances careful interpretation of results is required.

## Application of the *d*_*N*_/*d*_*S*_ Ratio Test to Plant Pathogens: Looking for Evidence of an Ancient Evolutionary Arms Race in Pathogen Lineages

### Tests Based on the *d*_*N*_/*d*_*S*_ Statistic May Be Difficult to Apply to Many Rapidly Evolving Effectors

Effectors, in the classic sense, are secreted pathogen proteins that suppress aspects of plant basal immunity. Detection of effectors by specific plant R genes elicits the secondary immune response, which prevents pathogen proliferation completely (Jones and Dangl, 2006). When recognized by R genes, effectors become Avr genes, as pathogen races that carry them are avirulent on hosts carrying their cognate R genes. This complete loss of virulence on hosts with specific R genes may exert very strong positive selective pressure on pathogen races that have lost corresponding Avr genes. Evidence of such selective pressure appears in many empirical observations. First, many effector genes lie in regions of the genome with many repeats and few other genes. This frequent observation among both fungi and oomycetes has been termed the ‘two-speed genome’ (Dong et al., 2015). Effectors in two-speed genomes lie within rapidly evolving regions with elevated transposable element activity. These tend to be far from the slow-evolving essential genes as most variation in essential genes is lost through purifying selection.

Effector sequences in fast evolving genome compartments can be easily duplicated, modified, inactivated, or lost entirely in a short space of time due to the activity of transposable elements. Presence/absence polymorphisms in particular are a major feature of many effectors. Take for example known *Avr* genes in *Magnaporthe oryzae*. Out of 164 isolates collected from various regions in the Philippines, between 5 and 81% contained each of the known Avr genes *Avr-Pik*, *Avr-Pita*, *Avr-Pii*, *Avr-Pizt*, and *Avr-Pia* (Lopez et al., 2019). The fact that none of these genes occur in all isolates suggests that loss of effector genes is a common response in *M. oryzae* populations to recognition by R genes. Duplication and diversification of existing effectors could be an evolutionary mechanism to replenish the pool of effectors after Avr genes are lost through negative selection. For example, in the powdery mildew pathogen *Blumeria graminis*, putative effectors and some known Avr genes occur in physically co-located gene clusters that have resulted from tandem duplications. The presence/absence of specific paralogous effectors is very variable between different *B. graminis* strains (Müller et al., 2019).

Host recognition of Avr genes in biotrophic pathogens provides a clear basis for strong positive selection. Isolates that are avirulent on the host simply do not survive and the population adapts. However, many necrotrophic pathogens produce other proteins, also described as ‘effectors,’ which promote cell death in plants. Recognition of such proteins leads to host susceptibility rather than resistance (Lo Presti et al., 2015). Loss of susceptibility loci in plants may make them less susceptible to pathogens with corresponding necrotrophic effectors (Faris et al., 2010). Although the strength of positive selection on isolates containing effectors that enhance pathogenicity is not well-studied, it seems that necrotrophic effectors may exhibit similar evolutionary characteristics to classic effectors. For example, presence of known necrotrophic effector-encoding genes *SnToxA*, *SnTox1*, and *SnTox3* is highly variable among 21 isolates of the wheat pathogen *Parastagonospora nodorum* (Syme et al., 2018). Such gene gains and losses could also be caused by activity of transposable elements as these genes are very close to genomic repeats (Syme et al., 2016).

**FIGURE 1:**
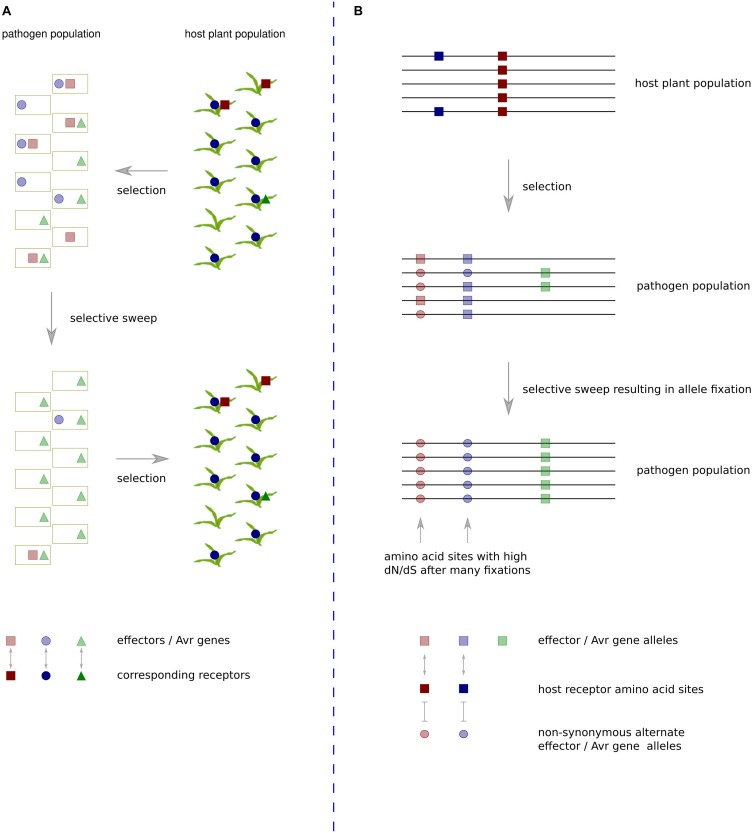
The trench warfare mode of pathogen × host co-evolution compared with a traditional arms-race scenario. **(A)** Under the trench warfare scenario, a pathogen population has a pool of rapidly evolving effectors, each cognate with different host receptors. The host plant population has a pool of rapidly evolving receptors, each cognate with different pathogen effectors. The complement of receptors in the host population exerts selective pressure on the pathogen population to lose specific effector sequences. If members of the host population frequently contain a receptor cognate with a particular effector, in this case the dark blue circles represent a frequently occurring host receptor cognate with an effector represented by light blue circles in the pathogen population, there will be strong positive selective pressure on pathogen isolates that do not contain the effector. The resulting pathogen population increases frequency of unrecognized effectors whilst losing the recognized effector. Such effectors exhibit a rapid turnover in repeat-rich genome compartments, and they often have no domains or detectable homologs in other species. **(B)** In the evolutionary arms race scenario, two specific loci interact, exerting strong selective pressure on particular amino acid sites in each other. In this example, the receptors of all host population members contain a site represented by a dark red square that recognizes a site in an effector of a pathogen population represented by a light red square. This recognition exerts strong selective pressure on members of the pathogen population that have undergone a non-synonymous mutation at that site to the light red circle allele. If selective pressure is maintained at a high level throughout the population, over time this will lead to fixation of the alternate allele. If the same sites continue interacting over generations, multiple amino acid fixations in the pathogen population will cause the sites to be detected by *d*_*N*_/*d*_*S*_ ratio tests as being under positive selection.

An evolutionary scenario involving rapidly changing heterogeneous effector protein complements and plant R or susceptibility gene complements has gained the description ‘trench warfare.’ This contrasts with the more traditional model of an evolutionary ‘arms race,’ which involves adaptation and counter-adaptation of two specifically interacting loci (Möller and Stukenbrock, 2017) (Figure 1). The former scenario is not always amenable to analyses based on the ω statistic as continual turnover of effector genes in fast-evolving genome regions often leads to development of species specific sequences that have no detectable homologs (Sonah et al., 2016). The latter scenario is amenable so long as sequences have not diverged too far between species. Although it seems many effector sequences evolve as part of a trench warfare system and have no homologs in other species, there are some examples with reliable enough homologs for application of ω based tests of positive selection. These will be discussed in the following section along with other pathogen proteins exhibiting evidence of positive selection at the codon level.

### Putative Effectors and Other Secreted Proteins Are a Notable Component of the Positively Selected Proteome

Secreted pathogen proteins, including effectors, are key players in interactions with hosts. Therefore, it is not surprising that they often exhibit evidence of past positive selection of non-synonymous mutations. In several species, secreted protein-encoding genes have more frequently experienced positive selective pressure than others have. Such is the case for the wheat pathogen *Zymoseptoria tritici*, which was analyzed in conjunction with two sister-species that infect wild grasses (Stukenbrock et al., 2011). In this species, proteins with a predicted secretion signal exhibited a higher average ω value than all other genes. Overall, it appears that such adaptive fixations were an important factor in the rise of *Z. tritici* as an aggressive pathogen of major economic significance. This is because adaptive fixations were likely more prevalent in *Z. tritici* than in its closest known relative, from which it diverged ∼11,000 years ago at the start of agricultural production in the Middle East.

In the smut pathogens *Ustilago hordei* and *Sporisorium reilianum* f. sp. *zeae* and *S. reilianum* f. sp. *reilianum*, genes encoding proteins with secretion signals were significantly enriched among genes with a high ω. Furthermore, compared with other genes, significantly fewer secreted protein encoding genes in the genomes of these fungi had homologs in other species. This could indicate rapid diversification of secreted proteins in a trench warfare model of evolution in conjunction with multiple adaptive amino acid changes in specific more conserved secreted proteins (Schweizer et al., 2018). These fungal species are known to produce biotrophic effectors that may succumb to recognition by plant R genes (Ali et al., 2014; Brefort et al., 2014), which aligns well with this mode of evolution.

Like the smut fungi, many rust-causing species in the subdivision Pucciniomycotina interact with plant hosts via classic biotrophic effectors (Petre et al., 2014). In a study by Sperschneider et al. (2014), the genomes of numerous diverse fungi were grouped using an unsupervised clustering algorithm based on sequence properties such as presence or absence of a secretion signal and amino acid composition. A set of 10,213 alignments of genes from only the Pucciniomycotina lineage with orthologs in three or more species were then scanned for signatures of positive selection using PAML. Out of the 387 genes with high site-specific ω values, 341 (88.1%) were in pathogen-specific gene families. One of the fungal sequence clusters identified contained predominantly small, cysteine-rich secreted proteins. Of the Pucciniomycotina genes with evidence of positive selection, 14 were in this cluster (Sperschneider et al., 2018).

In *Melampsora larici-populina*, which is also a biotrophic rust-causing fungus in the Pucciniomyctina subdivision, a large number of genes encoding paralogous predicted effectors exhibit evidence of positive selection. The residues in these proteins that appear to have undergone diversifying selection are predominantly located at the C-terminus, which suggests that this is a major site for interaction with host proteins. An interesting finding in this study is that many of the cysteine residues in these predicted effectors are evolutionarily constrained; these sites may be important for effector tertiary structure (Hacquard et al., 2012). Echoing this finding, the *Avr* gene *AvrP4* also shows evidence of positive selection across the *Melampsora* genus on residues in the C-terminus (Van der Merwe et al., 2009). In addition, it seems the RXLR effectors of the plant pathogenic oomycete genera *Phytophthora* and *Hyaloperonospora* have converged toward C-terminal positive selection. Overall, these effectors exhibit conservation at the N-terminus, which may be involved in secretion and host cell internalization, and adaptive evolution at the C-terminus (Win and Kamoun, 2008). Although distantly related to the fungi, the oomycetes in these genera establish similar biotrophic interactions with plants by producing effectors that silence host innate immunity. Again, these effectors can become detrimental to the pathogen when recognized by plant R genes (Du et al., 2018). The identification of many effector like proteins with enhanced ω values supports the hypothesis of an evolutionary regime that involves strong host-induced selective pressure on non-synonymous amino acid changes (Anderson et al., 2010). In a classic arms race scenario, the effector protein that is recognized by an R gene in the host would be under selective pressure to develop non-synonymous amino acid changes to avoid detection.

Evidence of ongoing positive selection of non-synonymous amino acid changes is found in *Z. tritici*. Several codons of the gene encoding the avirulence protein AvrStb6 may be positively selected due to their interactions with sites in the resistance protein Stb6 (Brunner and McDonald, 2018). In the hemibiotrophic rice pathogen *M. oryzae*, it would appear that multiple substitutions have affected the amino acid sequence of the gene *AVR-Pik*, which is normally recognized by the rice R gene *Pik*. A common haplotype of the *AVR-Pik* gene contains several mutations that make it resistant to all five known variants of *Pik* (Li et al., 2019). It is conceivable that such haplotypes in plant pathogens could become fixed before diversification of host R genes. This provides an evolutionary mechanism for the accrual of non-synonymous mutations in effector genes over time, which would show up in ω statistics for divergent sequences.

In addition to biotrophic effectors, some necrotrophic effectors also show signs of positive selection. A well-known group of necrotrophic effectors is the necrosis and ethylene inducing-like protein (NLP) family. In the plant pathogen genus *Botrytis*, which is composed of host-specific and broad host range necrotrophs, the two paralogous NLPs present appear to be under purifying selection at most sites. However, at two sites, there is evidence of a significant elevation in ω, indicating multiple fixations of non-synonymous polymorphisms in different *Botrytis* species (Staats et al., 2007). The significance of these sites in interaction with the host is unknown. Deletion of either of these proteins appears to have no effect on pathogenicity in *B. cinerea* (Cuesta Arenas et al., 2010). However, homologs of these genes have not been tested in any other *Botrytis* lineages and they do appear to elicit necrosis specifically in Dicotyledenous plants (Schouten et al., 2007). Therefore, it is certainly conceivable that the NLPs from other *Botrytis* lineages play a more pivotal role in infection, which has led to adaptive evolution in response to hosts.

Other host-facing proteins that exhibit evidence of positive selection are catabolic enzymes. For example, in three species of *Zymoseptoria*, 28 secreted proteases exhibit evidence of positive selection (Krishnan et al., 2018). The secreted proteases of plant pathogens are not especially well-characterized. However, some proteases may act as Avr proteins in biotrophic or hemibiotrophic fungi. For example, the *AVR-Pita* gene of the rice pathogen *M. oryzae* encodes a secreted peptidase that interacts directly with the *R* gene *Pi-ta* in the host; isolates carrying this secreted peptidase are avirulent on rice lines carrying *Pi-ta* (Jia et al., 2000). Alternatively, fixations of non-synonymous polymorphisms in these sequences could occur in response to subtle alterations in host protein substrates.

In addition to proteases, many plant pathogens secrete carbohydrate active enzymes (CAZymes). These enzymes play a crucial role in breakdown of the plant cell wall and appear to be more numerous among nectrotrophs than biotrophs (Lyu et al., 2015). A recent genome-wide scan for genes under positive selection among nine species of smut fungi identified several CAZymes with a high ω. The highest number of positively selected CAZymes in a single genome was in the fungus *Melanopsichium pennsylvanicum*. These five enzymes were all members of the esterase family, which contains many plant cell wall degrading enzymes. Thus, it is conceivable that during speciation, plant pathogens are subjected to positive selective pressure induced by changes in the carbohydrates present in plant cell walls. In some plants at least, specific cell wall components are associated with particular lineages (Sørensen et al., 2010), substantiating this hypothesis.

An alternative scenario that could lead to diversifying selection in secreted proteins is recognition by the plant innate immune system. The innate immune system of plants is geared toward detection of molecular motifs conserved throughout microbes, so called microbe associated molecular patterns (MAMPs). It is possible that proteins with conserved roles in pathogen viability could be recognized as MAMPs by plants. Major physiological functions among such proteins would lead to purifying selection across most residues. However, residues that lead to recognition by the host innate immune system might exhibit evidence of positive selection. This hypothesis was developed and tested on bacterial pathogens by McCann et al. (2012). This study identified 48 pathogen-specific conserved genes with evidence of dominant negative selection barring a few positively selected sites. Six of the proteins encoded by these genes elicited aspects of plant innate immunity. A similar study has not been performed in fungi but it may be a good starting point to test many of the positively selected secreted proteins identified in genome scans for elevated ω.

Some studies have shown the importance of genes other than secreted proteins in adaptation of plant pathogens. In a relatively early study, before genome sequence data were available, 42 genes were found with high ω values in the model biotrophic pathogen genus *Microbotryum*. Among these 42 genes, several were associated with intracellular processes that could be linked with specialization on different hosts. For example, there were several positively selected genes with domains involved in respiration under stressful conditions, and several with domains possibly involved in nutrient uptake from the host (Aguileta et al., 2010). In addition, besides the secreted protein-encoding genes under positive selection in the smut species *S. reilianum*, there were also many cytoplasmic protein-encoding genes. Many of these genes were annotated with Gene Ontology terms indicating an involvement in metabolism and cellular response to starvation. Notably, several Gene Ontologies associated with responses to external stimuli were enriched among positively selected genes in this species (Schweizer et al., 2018). Thus, tests for positive selection at the codon level may aid identification of numerous biologically important sequences other than candidate effectors and secreted proteins. Future studies could shift focus toward such genes as their functions are less explored in pathogens.

## A Brief Description of Some Methodologies for Performing Selective Sweep Scans

**FIGURE 2:**
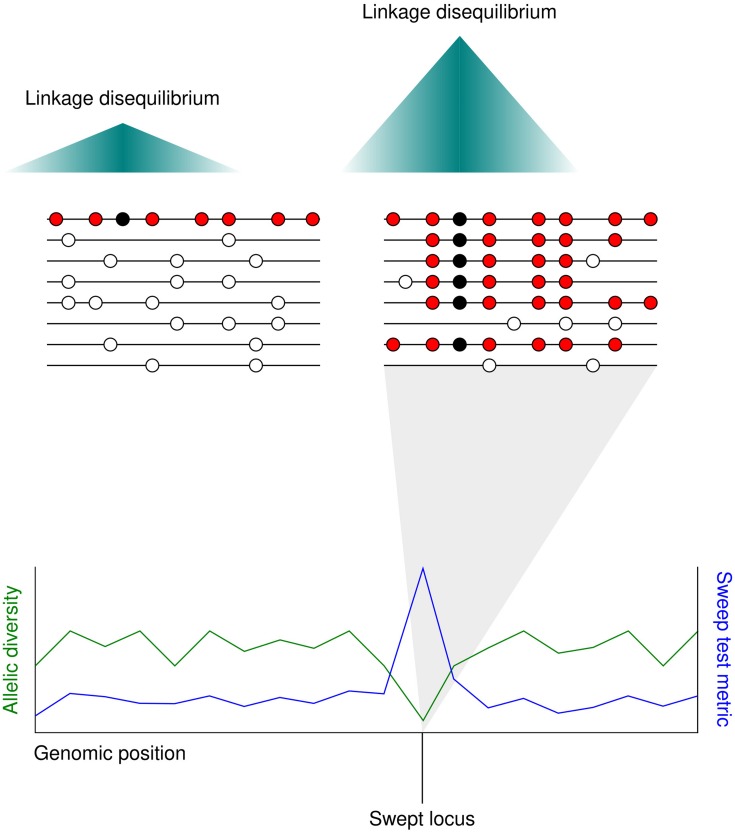
The localized allele frequency pattern following a selective sweep. **(Upper)** A favorable mutation (black circle) appears in an individual adjacent to several neutral polymorphisms (red circles). As the favorable mutation spreads through the population toward fixation the neutral alleles surrounding it also spread, replacing the neutral alleles in other individuals (white circles). The further from the beneficial mutation, the higher the chance of a neutral allele being lost through meiotic recombination. Since most individuals carry the same haplotype in the swept region, linkage disequilibrium is higher after the selective sweep. **(Lower)** Selective sweeps cause a localized drop in allelic diversity (green line). Whole genome scans for selective sweeps measure a statistic at different sites along the genome based on metrics such as local allelic diversity or long-range linkage disequilibrium (blue line).

The term ‘selective sweep’ applies to the fast spread of a haplotype containing a favorable allele through a population toward fixation. In sexually reproducing species, favorable loci undergoing selective sweeps may be distinguished from neighboring non-selected loci by a distinct drop in sequence diversity. The reason for this is that meiotic recombination separates chromosomal regions from each other over time. The loss in diversity surrounding the swept allele spans the extent of linkage disequilibrium in the species, as other alleles that are also neutral or weakly selected travel with the advantageous allele on the chromosome due to their physical proximity to it (Smith and Haigh, 1974) (Figure 2).

A simple test for positive selection in intraspecific sequences is analysis of Tajima’s D statistic (Tajima, 1989). This statistic is based on the nucleotide diversity (the average number of pairwise differences between individuals) of a sequence in a population, which is ascribed the Greek symbol ‘θ.’ An estimation of what θ should be under a completely neutral scenario including only genetic drift and mutation is obtained using knowledge of the number of polymorphic sites and number of individuals. This estimate is known as the Watterson estimator and is designated ${\hat{\theta }}_{w}$ (Watterson, 1975). Tajima’s D uses this estimate of θ in combination with a second statistic ‘*D*’; hence, Tajima’s ‘D.’ The *D* statistic is essentially an empirical measurement of the average number of pairwise differences between sequences. This can be compared to the expectation of this statistic given the number of individuals and the number of polymorphic sites, ${\hat{\theta }}_{w}$. If *D* is the same as ${\hat{\theta }}_{w}$, the null hypothesis that the sequence is evolving neutrally can be accepted. However, if it is lower, some unknown force has reduced the expected nucleotide diversity in the sequence. This force could be positive selection but it is important to note that deviations from ${\hat{\theta }}_{w}$ can also be caused by other factors such as changes in population size or gene flow with other populations. Therefore, Tajima’s D is rarely used in isolation and interpretation can be much improved if one uses allele frequency based inference of the demographic history of the population under study. Other early tests for deviations from neutral evolution include Fu and Li’s D and Fay and Wu’s H statistics (Fu and Li, 1993; Fay and Wu, 2000).

Such tests can be, and often have been, applied to whole genomes in sliding windows. This may help identify regions of decreased nucleotide diversity resulting from selective sweeps. However, several more sophisticated tests have been developed since the advent of whole genome sequencing and SNP genotyping. One popular method for identifying selective sweeps in whole genome data, called ‘SweepFinder,’ was developed by Nielsen et al. (2005). I will here provide a description of this test in plain language, which is not intended to be an in-depth explication of the theory but a contextual framework for the application examples later discussed.

First, the test defines a set of points along the chromosome of an arbitrary distance from one another; the more points there are the more coverage of the genome. Then, at each point, a function is maximized for a parameter denoted α. This parameter includes recombination, population size and selection strength and is used in conjunction with distance from the point in the genome to estimate the probability of an allele escaping a selective sweep occurring at that point. The test relies on the empirical allele frequency calculated across the whole chromosome for parameterization. In this way, it does not assume any specific model of DNA sequence evolution. Once the function has been maximized for alpha, the model’s log likelihood can be compared with the log likelihood of a model that does not allow for positive selective pressure at the locus to produce a composite likelihood ratio (CLR) statistic. The higher this statistic, the more evidence for a selective sweep at the given point along the genome. To determine whether the statistic produced by this test is significantly higher than expected for any site along the chromosome, simulated data must be produced with parameters approximating the demographic history of the sample under consideration. However, taking the 95^*th*^ percentile of observed CLRs may be acceptable as the test is more robust to demographic scenarios than those that rely on explicit models of sequence evolution.

In addition to the SweepFinder technique, several other related tests have been proposed. These techniques are reviewed and compared against one another in detail in Vatsiou et al. (2016). I will briefly describe the principles of these tests. Again, it is not my intention to provide an in-depth overview of the theory underlying these tests but to provide context for later examples of their application. The extended haplotype homozygosity (EHH) test was originally developed to scan for selective sweeps in the human genome (Sabeti et al., 2002). This statistic is based on the theory that new alleles with neutral impacts on fitness move slowly toward fixation due to genetic drift. The slow speed of this increase in frequency in the population leaves time for most neighboring alleles to be lost through recombination. However, a fast rise in allele frequency due to recent positive selection would lead to extended linkage disequilibrium between the allele and neighboring alleles. This is because the haplotype containing the favorable allele has spread too rapidly for the effects of linkage disequilibrium to separate it from neighboring loci (Figure 2). By measuring the linkage disequilibrium between a given haplotype and neighboring haplotypes, the EHH is designed to capture this signature of a recent selective sweep. Haplotypes with high frequency in the population and elevated long range linkage disequilibrium with neighboring haplotypes may be under positive selection. The test is limited to detection of recent selective sweeps as the swept haplotypes themselves will eventually lose neighboring alleles through recombination until they appear indiscriminate from fixed neutral mutations. The integrated haplotype score (iHS) test builds on the EHH test (Voight et al., 2006). The iHS score is an extension of the EHH that is normalized to the allele frequency at each site. Therefore, it is applicable across the whole genome regardless of allele frequency. This differs to the EHH as high EHH values should be combined with knowledge of the local allele frequency to identify positive selection. Secondly, when the iHS test was originally implemented, it was combined with knowledge of recombination hot and cold spots across the human genome. Extended homozygous haplotypes are more likely to occur in recombination cold spots, so the score is down-weighted in these regions; vice versa for recombination hotspots. The cross population EHH (XP-EHH) score was later devised by Sabeti et al. (2007). This method identifies haplotypes that have almost been swept to fixation in one sub-population but remain polymorphic in the population as a whole.

The tests described are sensitive enough to detect strong selective sweeps that have occurred recently. Strong selective sweeps that have not reached fixation tend to produce loci that have a single advantageous allele spread through most individuals in the population. This is known as a ‘hard’ selective sweep, distinguishing it from the alternative ‘soft’ selective sweep scenario. Under this scenario, the selective pressure is not very strong, or different alleles with equivalent benefits are selected from the standing genetic variation. This leads to a situation in which genetic diversity is reduced to a small number of equally advantageous haplotypes rather than a single predominant haplotype. The CLR, iHS, EHH, and XP-EHH tests are far less sensitive to detection of soft selective sweeps (Vatsiou et al., 2016). However, a recently developed program called S/HIC (Schrider and Kern, 2016, 2017) uses a machine learning algorithm and is reportedly sensitive to hard and soft sweeps and reliable in the face of population structure and demographic changes.

To test for significance of observed deviations in these statistics a demographic model can be fit to a SNP data set and its parameters used to simulate data arising under the same circumstances. A popular method for determining past demographic changes in populations using SNP data is the diffusion approach employed in the software package DaDi (Gutenkunst et al., 2009). This package comes in the form of a library of functions implemented in the programming language Python. It is therefore assumed that the user of this package has at least rudimentary knowledge of Python. The advantage of this implementation is its flexibility. Complex demographic models can be built with various different parameters influencing the allele frequency spectrum of the sample. Several reasonable models may be tested, for example, a population bottleneck followed by expansion, and compared with each other for fit to the data at hand using statistical bootstrapping techniques or likelihood ratio tests. Once a model with a relatively good fit has been found, its parameters may then be used to run a number of simulations in a coalescent simulator such as the program MS (Hudson, 2002). Typically, a threshold such as the 95^*th*^ percentile of test statistics observed from the simulations is used as a cut off for significance testing of candidate selective sweeps. Alternatively, taking the 95^*th*^ percentile of the empirical distribution of test statistics for the data at hand may be acceptable, especially when demographic inference is unreliable (Vatsiou et al., 2016).

## Application of Selective Sweep Tests to Plant Pathogens: Searching for the Genomic Footprints of Recent Adaptation

Although selective sweep scans have been performed on a large number of organisms, in particular model organisms and humans (Schlenke and Begun, 2004; Pritchard et al., 2010; Hernandez et al., 2011; Brand et al., 2013; Garud et al., 2015; Nam et al., 2017), there have been relatively few performed on plant pathogens. A common format for the studies performed on plant pathogens is a description of population sub-structure based on allele frequencies, analysis of population demographics, and, finally, detection of regions of the genome with significant evidence of positive selection. The following paragraphs describe the handful of studies on selective sweeps in plant pathogen genomes performed to date (Table 1).

**TABLE 1:** The five studies on selective sweeps in plant pathogens reviewed in this manuscript, including the minimum population size used for analysis.

References	Organism	Tests employed	Smallest population considered
Badouin et al., 2017	*Microbotryum* spp.	SweepFinder	17
Mohd-Assaad et al., 2018	*Rhyncosporium commune*	iHS | XP-EHH	14
Hartmann et al., 2018	*Zymoseptoria tritici*	SweepFinder |iHS | XP-EHH	24
Derbyshire et al., 2019	*Sclerotinia sclerotiorum*	SweepFinder	10

A study by Badouin et al. (2017) focuses on the two anther-smut fungus species *Microbotryum lychnidis-dioicae* and *M. silenes-dioicae*. In this study, 53 individuals are considered, including 34 *M. lychnidis-dioicae* and 19 *M. silenes-dioicae*. The 34 *M. lychnidis-dioicae* isolates were found to exhibit evidence of population substructure, which could bias inferences of positive selection. Therefore, the largest cluster among this group of 34, which consisted of 17 individuals, was used for selective sweep scans. Population demographic models were fit to the two populations to identify a threshold for significant detection of a selective sweep using the SweepFinder algorithm. In *M. lychnidis-dioicae* about 16–18% of the genome was found to be swept, whereas sweeps were present across only ∼ 1% of the *M. silenes-dioicae* genome. The ranges of percentages were calculated using different point spacings across the genome for the SweepFinder test. Most swept regions in this study contained multiple genes, which makes it difficult to draw any inferences linking gene function with positive selective pressure. However, several genes containing domains previously linked with pathogenicity in other plant pathogens were among those in swept loci. For example, genes encoding proteins containing “common in several fungal extracellular membrane proteins” (CFEM) domains were found in both species. The precise mechanisms of action of CFEM domain-containing proteins in plant pathogenesis are elusive. However, deletion strains for genes encoding them have shown pleiotropic phenotypes precluding full virulence on plants (Kulkarni et al., 2005; Zhu et al., 2017). Since they are secreted and anchored to the outer membrane, it is conceivable that they could interact with host proteins in some way. This provides a plausible explanation for the observation of candidate selective sweeps surrounding them.

A similar study was performed on the Barley scald-causing fungus *Rhyncosporium commune* by Mohd-Assaad et al. (2018). This study made use of a much larger sample than that of Badouin et al. (2017). A total of 125 isolates of *R. commune* were collected from geographically diverse locations spanning the globe. This larger set was divided into three genotypic clusters based on allele frequency data. Each of the three clusters were subjected to scans of selective sweeps using the iHS statistic. They were further compared with each other using the XP-EHH test. No demographic models were used and sites in either the 95^*th*^ or 99^*th*^ percentile of scores for each of the tests were chosen, depending on population size. The number of genes in swept regions ranged from 2 to 108 in this study. Again, this seems to preclude accurate inference of the functions of genes affected by swept alleles. However, in an attempt to achieve this, 5,000 bp regions either side of the two most significant SNPs in the genome were considered. These regions contained 15 genes, which included several with functional domains pertinent to plant pathogenesis. For example, several cell wall degrading enzymes were identified, as well as a multi-antimicrobial extrusion multidrug transporter. Such enzymes could be involved in direct interaction with plant substrates, which would provide a possible basis for positive selective pressure on alleles in their functional sites.

The wheat pathogenic fungus *Z. tritici* exhibits some characteristics that may make it highly amenable to detection of hard selective sweeps. It exhibits a pronounced race structure, with some isolates totally non-pathogenic on some cultivars depending on the presence or absence of different R genes. This makes populations very prone to very strong positive selective pressure when introduced to a host that they largely cannot infect. It also undergoes several propagation cycles a year, including a sexual phase; this allows for frequent meiotic recombination. Thus, any favorable alleles allowing the pathogen to propagate in a particular environment have a high chance of spreading sexually toward fixation. Accordingly, a study by Hartmann et al. (2018) showed many genomic regions with a pronounced CLR statistic. This study used a population sample of 123 isolates, which was subdivided into samples from specific globally distributed wheat fields that contained between 24 and 46 isolates each. This study implemented the SweepFinder algorithm and the iHS and the XP-EHH tests. As no demographic models were fit to the SNP data, the 99.5^*th*^ percentile of the CLRs was used to identify swept regions with the SweepFinder algorithm. Although this stringent threshold limited the investigation to just a few loci, it is notable that there were numerous CLR peaks throughout the genome that are much higher than the values used to call sweeps in the previously mentioned studies on *R. commune* and the *Microbotryum* species. There was some correspondence between the CLR and iHS based scans, which both produced notably high scores in some regions. In this study, the average number of genes found in a swept region was only six, which seems a reasonable number for follow-up wet-lab characterization. Among genes in these regions were many membrane-embedded transporters, secreted proteins and secondary metabolite biosynthesis genes. Remarkably, the Avr gene *Stb6* contained a SNP with strong evidence of population-specific positive selection. Thus, many expectations of the kinds of genes under host-induced positive selective pressure were met.

Finally, a study by my colleagues and I (Derbyshire et al., 2019) was recently performed on the broad host range necrotrophic plant pathogen *Sclerotinia sclerotiorum*. We performed Tajima’s D tests in sliding windows and CLR tests along the genome in two genotypically distinct pathogen populations. We found that there was no strong evidence for positively selected sites in the genome. This is quite striking as the CLR test was performed in exactly the same manner as on *Z. tritici*. One possible explanation for the lack of evidence for selective sweeps in *S. sclerotiorum* is its reproductive mode. This species is homothallic, thus it is self-compatible. Although it does show evidence of outcrossing, it seems that clonal reproduction is very frequent. This leads to very slow decay of linkage disequilibrium, which could confound the CLR test. Further, since *S. sclerotiorum* is able to infect many 100s of species, it may not be liable to the same strong selective pressures as species like *Z. tritici*. Host resistance to *Z. tritici* typically creates very strong selective pressure as the pathogen can easily become avirulent. This contrasts the situation in broad host range pathogens that may evolve more slowly toward optimization of metabolic functions (Badet et al., 2017). It is worth noting that we did find a single putative selective sweep that just made the threshold based on demographic inference. This sweep contained a single gene encoding a multidrug transporter. Although this domain may have some implications for pathogenicity, the finding is preliminary and further tests should be performed on this species to characterize its evolutionary dynamics.

## Conclusion

Scans of whole genomes for evidence of positive selective pressure have the potential to elucidate fundamental aspects of plant pathogen evolution as well as identify candidate genes for functional characterization. The two widely employed classes of tests that focus on the ω statistic or allele frequency spectrum at different genetic loci both identify cases of positive selection but apply to very different time scales. The ω test assumes multiple fixation events between sequences, and therefore describes an older signature of positive selection. A high ω among pathogen genes does not likely indicate adaptation to recent shifts in agricultural practices as the time frame is simply too small. However, knowledge of genes that have been a crucial part of a particular species’ adaptation may provide insights into the broader evolutionary context of plant pathogens. The selective sweep scan is amenable to detection of strong recent positive selective pressure in populations, which is highly applicable to plant pathogens in an agricultural context. However, care must be taken when interpreting the results of such analyses, as pathogen lifecycle, mode of reproduction and population history can strongly affect inference. Furthermore, identification of the precise allele under selective pressure remains elusive, especially for species with slow linkage disequilibrium decay. Therefore, phenotypic data and functional studies should also be considered to narrow down the search for genes important to pathogen adaptation. As more and more plant pathogen genomes are sequenced, analysis of positive selection pressure may become an important tool to identify genes involved in recent adaptation events such as fungicide resistance development or evasion of host R genes.

## Author Contributions

The author confirms being the sole contributor of this work and has approved it for publication.

## Conflict of Interest

The author declares that the research was conducted in the absence of any commercial or financial relationships that could be construed as a potential conflict of interest.
